# Analysis of Immunoglobulin Transcripts in the Ostrich *Struthio camelus*, a Primitive Avian Species

**DOI:** 10.1371/journal.pone.0034346

**Published:** 2012-03-29

**Authors:** Tian Huang, Min Zhang, Zhiguo Wei, Ping Wang, Yi Sun, Xiaoxiang Hu, Liming Ren, Qingyong Meng, Ran Zhang, Ying Guo, Lennart Hammarstrom, Ning Li, Yaofeng Zhao

**Affiliations:** 1 State Key Laboratory of Agrobiotechnology, College of Biological Sciences, National Engineering Laboratory for Animal Breeding, China Agricultural University, Beijing, People's Republic of China; 2 College of Animal Science and Technology, Henan University of Science and Technology, Henan, People's Republic of China; 3 Division of Clinical Immunology, Department of Laboratory Medicine, Karolinska University Hospital Huddinge, Stockholm, Sweden; 4 Key Laboratory of Animal Reproduction and Germplasm Enhancement in Universities of Shandong, College of Animal Science and Technology, Qingdao Agricultural University, Qingdao, People's Republic of China; National Institute on Aging, United States of America

## Abstract

Previous studies on the immunoglobulin (Ig) genes in avian species are limited (mainly to galliformes and anseriformes) but have revealed several interesting features, including the absence of the IgD and Igκ encoding genes, inversion of the IgA encoding gene and the use of gene conversion as the primary mechanism to generate an antibody repertoire. To better understand the Ig genes and their evolutionary development in birds, we analyzed the Ig genes in the ostrich (*Struthio camelus*), which is one of the most primitive birds. Similar to the chicken and duck, the ostrich expressed only three IgH chain isotypes (IgM, IgA and IgY) and λ light chains. The IgM and IgY constant domains are similar to their counterparts described in other vertebrates. Although conventional IgM, IgA and IgY cDNAs were identified in the ostrich, we also detected a transcript encoding a short membrane-bound form of IgA (lacking the last two C_H_ exons) that was undetectable at the protein level. No IgD or κ encoding genes were identified. The presence of a single leader peptide in the expressed heavy chain and light chain V regions indicates that gene conversion also plays a major role in the generation of antibody diversity in the ostrich. Because the ostrich is one of the most primitive living aves, this study suggests that the distinct features of the bird Ig genes appeared very early during the divergence of the avian species and are thus shared by most, if not all, avian species.

## Introduction

The adaptive immune system of jawed vertebrates is characterized by the production of immunoglobulins (Igs) in response to antigens [1]. The B cell antigen receptor Ig is a heterodimeric protein that is usually composed of two identical heavy (H) chains and two identical light (L) chains. A disulfide bond formed by cysteine residues between the C_L_ and C_H_1 domains covalently joins the L chain to the H chain, and the two V domains associate non-covalently to form the antigen-binding site [2].

The Ig classes (in mammals, IgM, IgA, IgD, IgG and IgE) are defined by the isotypes of the heavy chain constant genes (μ, α, δ, γ and ε). Additional Ig isotypes have been identified in lower jawed vertebrates, including birds, reptiles, amphibians, bony fish and cartilaginous fish [3]. IgM is structurally conserved throughout evolution and is expressed in all jawed vertebrates. IgD is as ancient as IgM and has been described in elasmobranchs (in which it was previously known as IgW), bony fish, amphibians, reptiles and mammals [4], [5], [6], [7], [8]. IgD is, however, absent in birds and several mammals such as rabbits, opossum and elephants [9], [10], [11], [12]. Compared with IgM, IgD shows a high degree of structural plasticity because of variance in the copy number and number of C_H_ encoding exons as well as alternative RNA splicing [13]. In addition to these ancient Ig classes, some additional distinct Ig classes have been found in different vertebrates, such as IgY in lower tetrapods [7], [9], [14], IgNAR in cartilaginous fish [15], IgT/IgZ in the trout and zebrafish [16], [17], IgX and IgF in amphibians [6], [18], and IgO in the platypus [19].

The L chains contribute considerably to combinatorial antibody diversity by their association with H chains [20]. It is known that cartilaginous fish, teleost fish and amphibians express three IgL isotypes: κ, λ and σ [21], [22], [23]. A fourth IgL isotype, σ-cart, is only found in sharks [24]. Evolutionarily, fewer types of Ig light chains are present in mammals and reptiles, which express only λ and κ. The two light-chain loci differ significantly in their genomic organization. At the λ locus, multiple Vλ segments are followed by Jλ-Cλ repeats. By contrast, the κ chain–encoding locus contains only a single Cκ gene with a small cluster of Jκ and multiple Vκ genes located upstream [25], [26], [27]. Surprisingly, birds exclusively express λ light chains [28], [29]. The chicken and zebra finch IgL loci include only one functional IGVL gene and one IGJL gene, but multiple IGVL pseudogenes are located upstream of this functional IGVL gene [30], [31]. Light chain diversity is generated by intrachromosomal gene conversion using the upstream pseudo-Vλ gene segments as donor sequences [32].

The avian species described to date express only three immunoglobulin classes: IgM, IgA and IgY, which are encoded by Cμ, Cα and Cυ respectively [33], [34], and no IgD encoding gene has been identified. The Cυ and Cα genes in the chicken and duck IgH loci are positioned in reverse orientation [14], [35], which raises questions regarding the mechanism of class switch recombination in birds and the evolution of the IGHC gene locus. IgY is a monomeric antibody of low molecular weight found in amphibians, reptiles, and birds and is thought to be the ancestor of mammalian IgG and IgE [36]. In addition to the full-length IgY, ducks can also generate a truncated IgY termed IgY(ΔFc), which is expressed by the alternative transcriptional termination of the single υ gene [37], [38].

Birds represent an enormously diverse group of vertebrates comprising nearly 9000 species. Our knowledge of the avian Ig genes is currently restricted to a few galliform (chicken, turkey, pheasant and quail) and anseriform birds (duck) [34]. According to phylogenetic studies, these two groups of birds diverged only recently (approximately 100 million years ago [MYA]) [39]. The ostrich (*Struthio camelus*) belongs to the ratitae order, which represents the most primitive living aves, i.e., birds that diverged from other avian lineages approximately as early as 140 MYA [40]. In the present study, we analyzed the Ig genes in this species to investigate whether it expresses other Ig isotypes in addition to the IgM, IgA, IgY and λ light chains. Our objective was to provide additional clues to understand the evolution of Ig genes in birds.

## Results

### IgH classes expressed in the ostrich

**Figure 1:**
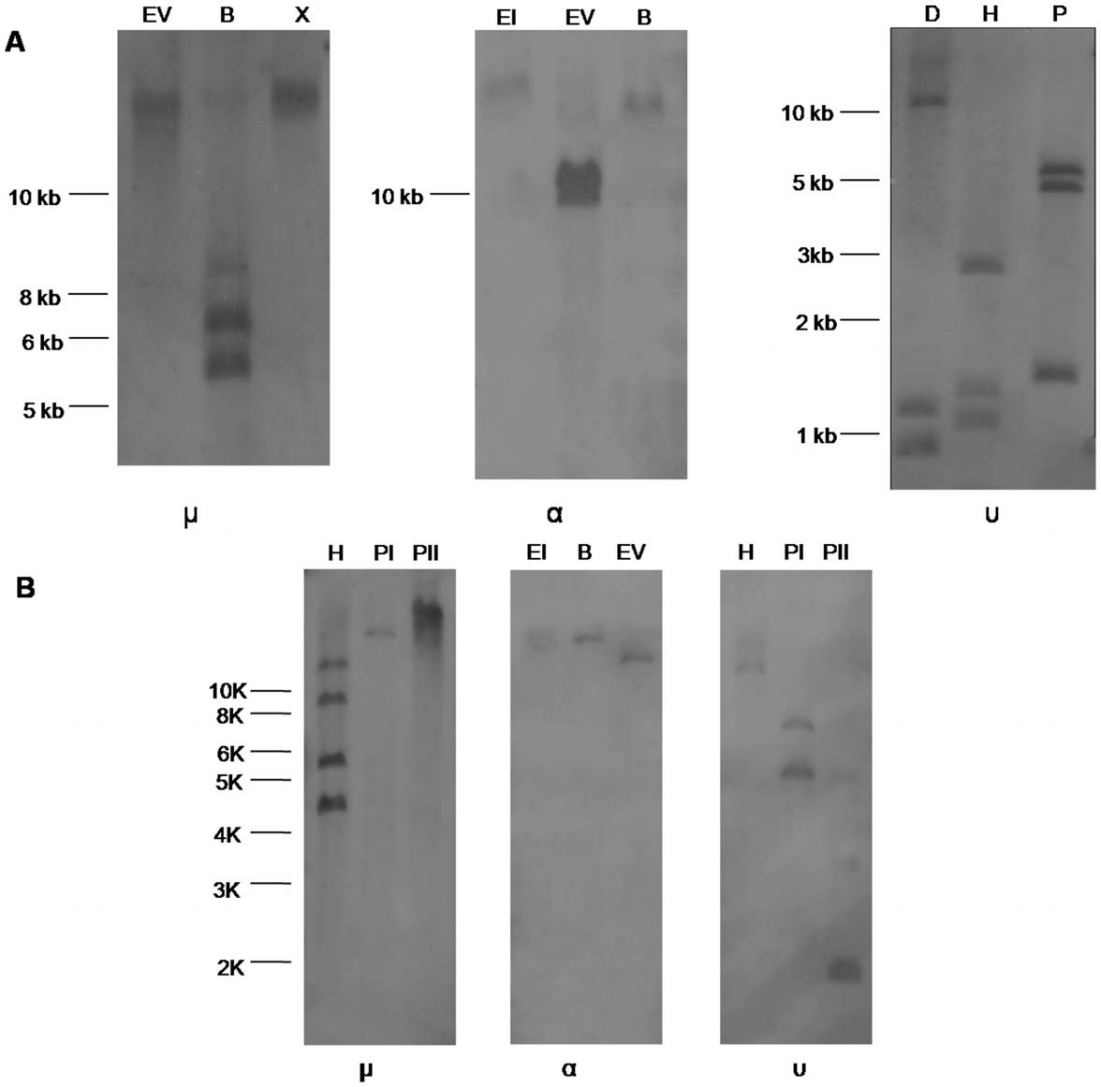
Southern blot detection of the ostrich Ig heavy chain constant-region genes. A. Southern blot detection of the ostrich Ig heavy chain constant region genes using Cμ-, Cα-, and Cυ-specific full-length probes. EI, *Eco*R I; EV, *Eco*R V; B, *Bam*H I; X, *Xb*a I; D, *Dra* I; *H, Hin*d III; P, *Pst* I. B. Southern blot detection of the ostrich Ig heavy chain constant region genes using Cμ4, Cα3, Cυ4 single-exon probes. EI, *Eco*R I; EV, *Eco*R V; B, *Bam*H I; *H, Hin*d III; PI, *Pst* I; PII, *Pvu* II.

To analyze the IgH classes expressed in the ostrich, we generated two Ig-specific mini-libraries using the total RNA isolated from the spleen and intestine. In total, 234 clones derived from the spleen library were analyzed. Most clones (199) were found to contain IgA cDNA, whereas only 16 IgM- and 5 IgY-containing clones were identified. The remaining 14 clones were shown to contain non-Ig sequences. It is surprising that the IgA clones comprised such a large portion of the library. This finding is likely the result of PCR bias in the construction of the library. We only identified IgA clones (319 clones) from the intestine library (total of 327 clones analyzed). These data suggest that the ostrich also expresses IgM, IgY and IgA, similar to chickens and ducks. The presence of these encoding genes in the ostrich genome was subsequently confirmed by Southern blotting using Cμ-, Cα- and Cυ-specific full-length probes (Fig. 1A).

To investigate whether the ostrich expresses IgD, we designed several pairs of degenerate primers based on the conserved Cδ regions of other species. However, we did not to amplify any putative IgD sequence regardless of whether cDNA or genomic DNA was used.

### Analysis of the ostrich Cμ gene

Analysis of the obtained IgM heavy chain constant-region cDNA clones revealed only a unique sequence, which suggests the expression of a single μ gene. However, four bands were detected when the μCH4 sequence (containing no *Hin*d III site) was used as a probe in the Southern detection of *Hin*d III-digested genomic DNA (Fig. 1B), which indicates the presence of more than one μ genes in the ostrich genome.

**Figure 2:**
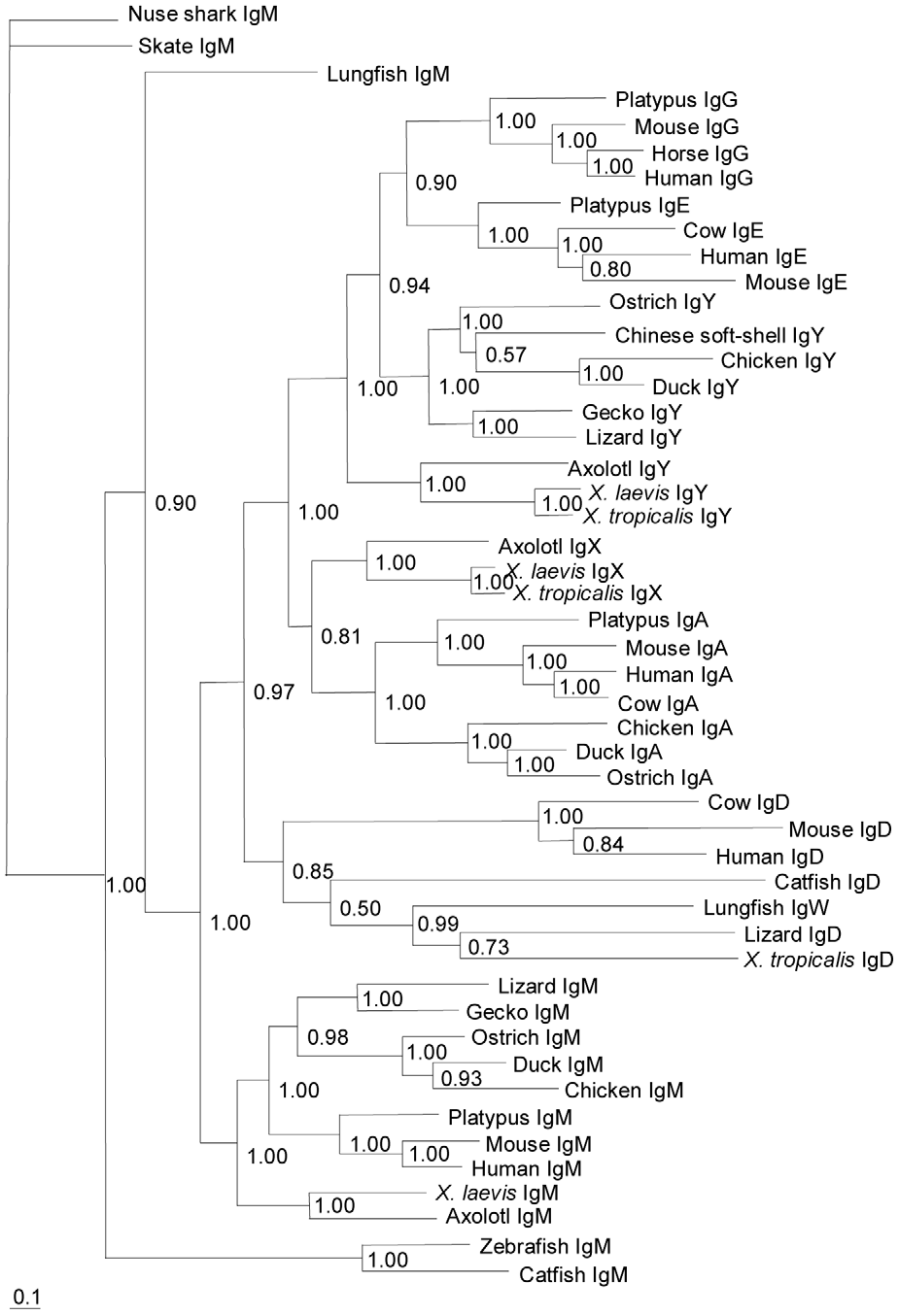
Phylogenetic analysis of the ostrich immunoglobulin genes. The amino-acid sequences of all C_H_ domains were used for the tree construction. For those species that express IgD (and IgW) longer than four CH domains, only the first four CH domains were used in the tree construction. The scale bar represents the genetic distance. The credibility value for each node is shown.

The obtained ostrich secretory IgM heavy chain constant-region cDNA encodes 447 amino acids, in which 12 cysteines are positionally conserved compared with Cμ in other species (Fig. S1). All of the aligned Cμ sequences (secreted form) exhibit an identical three-amino-acid motif (TCY) in their carboxy terminals (Fig. S1), which is where the cysteine is assumed to bind the J chain to form polymeric IgM [41]. The entire ostrich IgM constant region contains four potential N-linked glycosylation sites (N–X–S/T): N-46, N-127, N-199 and N-434. Only N-46 and N-434 are conserved among reptiles, birds and mammals [42], [43]. The N-127 site is conserved in birds and reptiles. The N-199 site is found exclusively in birds (Fig. S1). Alignment of the ostrich IgM constant region with those of other species demonstrated that the Cμ3 and Cμ4 domains to be less divergent than the Cμ1 and Cμ2 domains (Fig. S1). The ostrich IgM constant region shares an overall identity of 53.1% and 63.1% with the chicken and duck Cμ, respectively, at the protein level. The identity of the ostrich IgM is supported by a phylogenetic analysis (Fig. 2).

**Figure 3:**
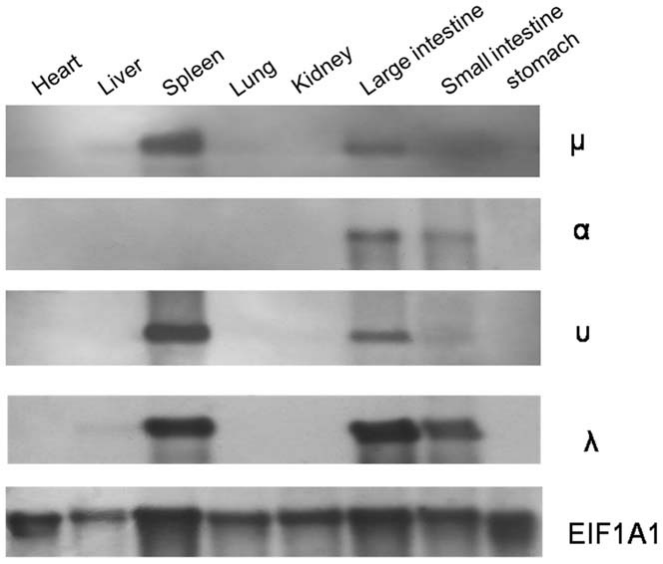
Northern blot analysis of ostrich μ, α, υ and λ gene expression in different tissues.

Northern blotting to detect IgM gene expression further showed that the ostrich μ gene was primarily expressed in the spleen and large intestine and only weakly expressed in the liver and small intestine (Fig. 3) although RT-PCR showed IgM transcripts to be present in all tissues examined (Fig. S2).

### Analysis of the ostrich α gene

Southern blotting with either the full-length or Cα3 exon as probes suggested that a single α gene was present in the ostrich genome (Fig. 1). IgA is the principal antibody class in mucosal secretions and acts as an important first line of defense [44]. It is usually highly expressed in mucosal tissues but only weakly expressed in the spleen. However, most clones in our spleen-derived Ig-specific mini-library were found to be IgA. This finding could be the result of a PCR bias during the process of 3′ RACE. Indeed, our RT-PCR and Northern blotting data showed that the ostrich IgA was primarily expressed in the large and small intestines (Fig. S2, Fig. 3).

When comparing the ostrich IgA heavy chain constant region with those of other species, 10 conserved cysteines were observed. There are three N-linked glycosylation sites in Cα2, Cα3 and the canonical secretory tail: N-165, N-221 and N-419, all of which are conserved in birds (Fig. S3). The ostrich Cα gene shares 44% sequence identity with chicken and 66% with duck Cα.

**Figure 4:**
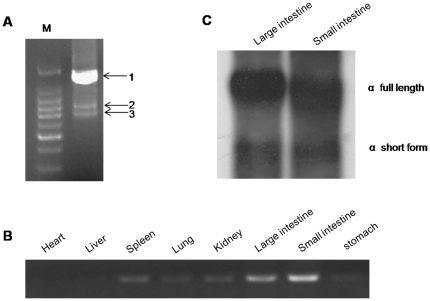
PCR and Northern blot analysis of IgA gene expression. A. PCR product of IgA 3′RACE. M, 100-bp DNA marker; 1, full-length IgA form; 2, non-specific band; 3, short form of IgA. B. RT-PCR detection of the short form of transmembrane IgA gene expression in different tissues. C. Northern blot analysis of ostrich full length and short-form of IgA gene expression in different tissues.

When performing 3′RACE PCR using the spleen RNA and J_H_-derived primers, we observed an 850-bp band in addition to the major 1.6-kb products (all 4-Cα containing transcripts). Sequencing of this band showed that it encoded a short, membrane-bound IgA lacking the last two Cα domains (i.e., VDJ-Cα1-Cα2-TM) (Fig. S4). To further confirm the presence of this short transcript, we used primers derived from the Cα1 to perform IgA-specific 3′RACE. In addition the full-length of 1.4-kb IgA transcript, we again detected the short IgA transcript, which contained only the first two Cα domains (Fig. 4A). To determine whether the short IgA is only expressed in the spleen, we then performed RT-PCR using the primers derived from the Cα1 and TM regions. The short form was detected in multiple tissues (Fig. 4B). Northern blotting with the first two Cα exons as a probe showed the short form to be mainly expressed in the intestine, albeit at a much lower level than the full-length form (Fig. 4C). To confirm that the short IgA transcript was derived from alternative splicing, we amplified and sequenced the exon-intron boundaries of Cα2-intron-Cα3, and Cα4-intron-TM, which clearly demonstrated that the short form to be derived from splicing of the Cα2 onto the TM exon.

**Figure 5:**
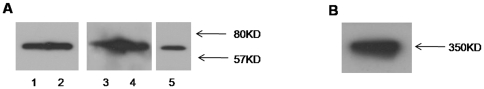
Western blot detection of ostrich IgA. A. IgA expression in tissues and secretions. 1, Large-intestine cytoplasmic proteins; 2, Small-intestine cytoplasmic proteins; 3, Cell membrane proteins of the large intestine; 4, Cell membrane proteins of the small intestine; 5, Large-intestine secretions. B. Dimerized IgA detected in large-intestine secretions.

The presence of the short IgA transmembrane transcript raises a question as to whether the ostrich is able to express a secreted IgA form lacking the last two Cα domains (i.e., IgA(ΔFc), similar to IgY(ΔFc) in ducks), although we did not observe such transcripts in the RACE experiments. We thoroughly analyzed the intron sequence between Cα2 and Cα3, and did not find any potential transcriptional termination signal or polyadenylation signal sequence (i.e., AATAAA). A polyclonal rabbit antiserum against the ostrich Cα1 and Cα2 were used in Western blotting. Only the intact form of IgA (approximately 65 KD under reducing conditions) was detected in the intestine membrane and cytoplasmic proteins (Fig. 5A). No short form of IgA could be identified at the protein level, probably because of an extremely low level of expression. The IgA in secretions of the large intestine appeared to be dimeric (approximately 350 KD under non-reducing conditions), as under reducing conditions, the molecular weight of the IgA heavy chain (without light chains) is approximately 65 KD (Fig. 5B).

### Analysis of the ostrich υ gene

The full-length IgY heavy chain constant region cDNA (secreted form) was obtained by screening the spleen Ig-specific mini-library. A phylogenetic analysis indicated that it was the ostrich υ gene (Fig. 2). Similar to ostrich μ, we only obtained a single IgY heavy chain constant-region cDNA, although Southern blotting indicated that more than one υ gene was present in the ostrich genome (Fig. 1). Alignment of the ostrich IgY heavy chain constant region with those of other species revealed two cysteines in the Cυ1, which suggests that these molecules can associate with light chains. Seven additional cysteines were distributed in Cυ2-Cυ4, all of which are conserved across all species examined (Fig. S5). Cυ contains two N-linked glycosylation sites: N-166 in the Cυ2 conserved in birds and lizards and N-265 in the Cυ3 conserved in *Xenopus* and humans (Fig. S5). A domain-by-domain comparison of the Cυ regions indicated that the Cυ1 displayed the lowest amino acid identity in birds (Fig. S5).

The expression pattern of the ostrich IgY transcript was examined using RT-PCR and Northern blotting suggested that the υ gene was primarily expressed in the spleen and large intestine (Fig. S2, Fig. 3).

### Analysis of rearranged VDJ fragments

To analyze the expressed VDJ sequences, 5′RACE was performed using the primers derived from the μ, α and υ chain constant regions. The inferred amino acid sequences were aligned and showed relatively low sequence diversity. The amino acid sequence variabilities of the V_H_ region were mostly confined to the CDR regions, in particularly the CDR3 region [45]. We sequenced 83 cDNA fragments, which provided 54 unique CDR3 (Fig. S6). The length of CDR3 varies from 9 to 24 residues to create considerable variability with an average of 14.33±2.18 codons, which is longer than the CDR3 of *Xenopus* (8.6 codons) and mice (8.7 codons) [46]. Analysis of the FR4 sequences suggests that there are two distinct J_H_ gene segments in the ostrich: J_H_1 and J_H_2, which differ by seven nucleotides but have only one amino-acid substitution (Fig. S7). Among the obtained V_H_ clones, more than 10 contained leader peptide-encoding sequences that were identical in sequence (MGPRLPGFVLLLLLLAALPGLRA). It is highly likely that only a single V_H_ gene segment was available for VDJ recombination events in the ostrich.

### Analysis of the ostrich light chain genes

**Figure 6:**
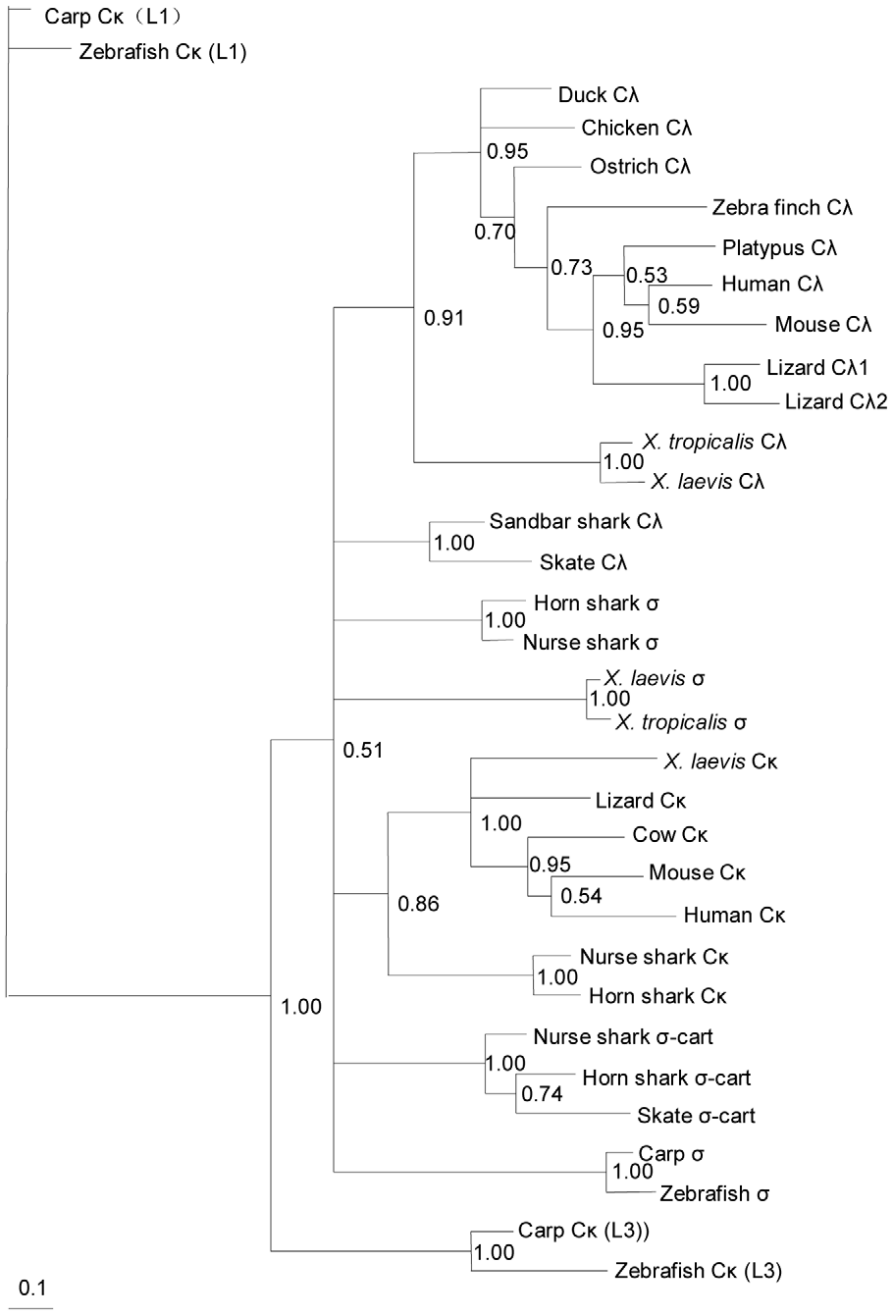
Phylogeny of the IgL chain constant-region genes in jawed vertebrates. The amino acid sequences were used for the tree construction.

To analyze the light chain genes in the ostrich, we designed several pairs of degenerate primers for the λ and κ genes based on the conserved Cλ and Cκ sequences of other species. These primers were used in PCR amplifications with the spleen cDNA as templates. We were only able to amplify the λ gene in the ostrich, as a phylogenetic analysis clearly showed that the identified gene belonged to the λ lineage (Fig. 6). We further performed 5′ RACE amplifications based on the Cλ sequence that we obtained. In total, 57 clones were sequenced and shown to encode the full-length Vλ domain and the same leader peptide (MAWAPLLLAVLAHGSGSLV). Overall, the inferred Vλ region amino acid homology among these clones ranged from 87.2 to 99.2%. The average length of the CDR3 was 9.81±2.38 codons, with a range of 4 to 14 codons. The tetrapod IGVL sequences generally have a fairly well-conserved DEAD (Asp–Glu–Ala–Asp) motif in the FR3 region [47]. However, as in the chicken IGVL sequence (DEAV), the Asp residue is also substituted by a Val in the ostrich. An analysis of these Vλ sequences revealed only a single Jλ in the ostrich.

We subsequently performed 3′RACE using the leader peptide specific primers and which identified a single Cλ in the ostrich. The Cλ sequence shows a 67.0%, 55.1% and 64.5% amino acid sequence homology to the chicken, lizard and human Cλ1, respectively. A protein sequence alignment of Cλ in amphibians, reptiles, birds and mammals revealed an identical pattern with regard to the cysteine distribution (Fig. S8).

## Discussion

**Figure 7:**
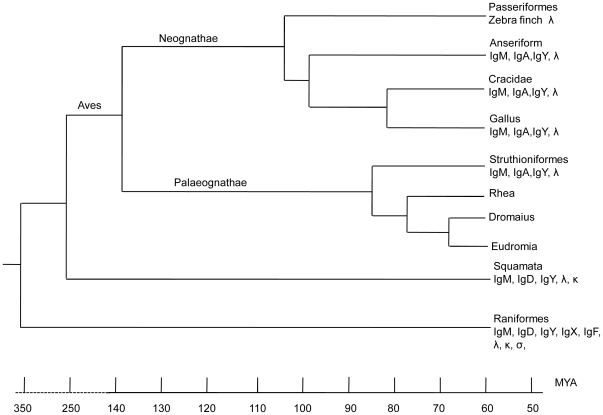
Phylogeny of the Ig isotypes in birds. (Only the λ light chain gene has been analyzed in zebra finch [Passeriformes]). The divergence time of the bird lineages was based on three references [40], [48], [52].

In the present study, three Ig isotypes (IgM, IgA and IgY, but not IgD nor Igκ), were identified in the ostrich, which is a primitive avian species belonging to the order struthioniformes. Although Southern blotting indicated that there was more than one copy of the μ and υ genes in the ostrich genome, we were only able to obtain one copy of the μ and υ expressed at the cDNA level. We amplified and sequenced the intron between Cμ1 and Cμ2 and obtained only a single sequence. We also performed PCR using two pairs of primers derived from the conserved Cυ4 sequences using genomic DNA. All sequenced clones were shown to contain the same sequence. The additional μ and υ genes detected by Southern blotting were likely pseudogenized and have diverged. We also performed Southern blotting using probes for the IgD and Igκ constant regions of crocodiles and could not detect any bands (data not shown). It is likely that, as in chickens and ducks, the δ and κ genes are both absent in the ostrich; however a definite conclusion cannot currently be reached. All of the expressed ostrich heavy chain and light chain V regions harbored the same signal peptide, which indicates that there is only one functional V_H_ and Vλ involved in V(D)J recombination in the ostrich. It is reasonable to assume that the ostrich also uses gene conversion as a major mechanism for generating antibody diversity. The ostrich Ig genes essentially exhibit the same distinct features that have been previously observed in chickens and ducks. This similarity demonstrates that the typical bird Ig system was likely already present in the common ancestor of carinatae and ratitae bird species and has remained unchanged over a long period of evolution (Fig. 7).

Reptiles are the closest relatives of the aves, and they are believed to have diverged approximately 250 MYA [48]. Recent studies have shown that reptiles, such as lizards and turtles, express IgD and κ light chains [7], [27], [49]; this finding suggests that the evolutionary loss of these two genes must have occurred in birds after their divergence from reptiles (Fig. 7). Another interesting issue regarding IgA evolution also arises when considering findings present in both reptiles and birds. The IgA-encoding gene in ducks and chickens shows a transcriptional orientation opposite to that of IgM and IgY [9], [14]. We also recently showed that the IgA encoding gene was absent in lizards and some other reptiles ([7] and our unpublished data), which suggests that the IgA gene in the lineages leading to reptiles and birds has undergone some gene rearrangements that either deleted or inverted this gene. These germ-line DNA rearrangements in the IgH locus might also account for the evolutionary loss of the IgD gene in birds. A future investigation on the Ig genes in more primitive living birds or reptiles may help to clarify this issue.

When analyzing the ostrich IgA transcripts, we identified a shorter membrane-bound IgA encoding form with the last two Cα exons removed, as the Cα2 exon was directly spliced onto the TM exon. However, this short form of IgA could not be detected at the protein level, which suggests limited to no functional significance. Indeed, this short form of the IgA transcript was present at a very low level even at the mRNA level, and its presence may simply be to the result of accidental RNA splicing caused by non-critical mutations around the splice sites.

In summary, we characterized three Ig heavy chain classes (IgM, IgA and IgY) and the λ light chain in the ostrich in this study. This study enriches the current knowledge of ratitae Igs, provides support for the continuous evolution of immunoglobulins in birds and highlights the importance of comparative studies in understanding the evolutionary history of the immune system.

## Materials and Methods

### Animals, RNA and DNA isolation

Ostriches (*Struthio camelus*) were purchased from a local Beijing farm. The animals were treated in accordance with the guidelines of China Agricultural University regarding the protection of animals used for experimental and other scientific purposes. The study was approved by the ethics committee of China Agricultural University (ID number 20110302). The total RNA was extracted from different tissues using the TRNzol kit (TianGen Biotech), following the manufacturer's instructions. Genomic DNA was extracted from the liver following routine protocols.

### Construction and screening of spleen and intestinal Ig-cDNA libraries

Approximately 2 µg of spleen and intestine total RNA was used to synthesize first-strand cDNA with a First-Strand cDNA synthesis kit (Promega, USA). One pair of degenerate primers, V_H_s (5′-CCH RGV AAG GGG CTS GAG TGG GT-3′) and IgAas (5′-TTG ACW TKG GTG GGT TTA CC-3′), was designed based on the conserved V_H_ and IgA C_H_ regions. The products were gel-extracted, ligated into the pMD19-T vector (Takara) and sequenced. J_H_-specific primers were designed according to the analysis results. First-strand cDNA synthesis was performed with *Not* I-d (T) 18 primers (5′-AAC TGG AAG AAT TCG CGG CCG CAG GAA TTT TTT TTT TTT TTT TTT-3′). J_H_1s (5′-GCC GGG GCA CCT CGG TCA CCG TCT CCT CA-3′) and J_H_2s (5′-GCC GCG GGA CGG CCG TCA CCG TGT CCT CA-3′) were mixed and used as sense primers for one round of 3′RACE PCR for the ostrich heavy chain constant regions. The resulting 1.6-kb PCR products were cloned into a T vector to generate an Ig cDNA mini-library. The white clones (after blue-white screening) were subjected to PCR screening using the universal primers M13F (5′-GCT TCG AAT GTA AAA CGA CGG CCA GT -3′) and M13R (5′-GCT TCG AAC AGG AAA CAG CTA TGA C -3′) for positively recombined clones containing the correct insert size; IgM1 (5′-CGT CAA CGA CAG CAT CTC CA -3′) and IgM2 (5′-GCG AGC ACC AGG GAC ACA TT-3′) for IgM-positive clones; IgA1 (5′-GCG CTC TGG ACG TGA CCT CCG A -3′) and IgA2 (5′-GAC GCT CAG CTT GCT GTA GAC -3′) for IgA-positive clones; and IgY1 (5′-CTG CCT CAT CTC CCA CTT CTA C-3′) and IgY2 (5′-TCT CGG TGC AGT GGC TCA AGA AC-3′) for IgY-positive clones. The clones that contained correct insert size but were negative for IgM, IgA and IgY were sequenced to identify the inserts. The primers used for cloning the ostrich V_H_ region were MGSP1 (5′-CTG GAG ATG CTG TCG TTG ACG TAG T-3′), MGSP2 (5′-TGA CGT AGT TGG TCC AGG AGA A-3′), AGSP1 (5′-CCG GGT AGG TCA AGA CCT CTG A-3′), AGSP2 (5′-TCG CTG GAA ACC CAG GTG AC-3′), YGSP1 (5′-TGG CGG CCA TCG CAA ACT GGC T-3′), and YGSP2 (5′-TAG AGG CCG GAG CGG AAG AG-3′); all of the primers were designed from the C_H_ regions that we obtained. The PCR experiments were performed according to the instructions of the 5′-RACE System for Rapid Amplification of cDNA Ends (Invitrogen, USA). The 650-bp PCR products were cloned into the pMD19-T vector and sequenced directly. Similarly, the ostrich Ig light-chain spleen and intestinal cDNA min-libraries were constructed and screened. The primers used to amplify the Igλ constant region were CLs (5′-AAH AAG GCC ACM CTG GTG TG-3′) and CLas (5′-CAG GTA RCT GCT GDC CAT RTA-3′). Primers LGSP1 (5′-GTA CTG GTT GTT GCT CTG-3′) and LGSP2 (5′-CCA CAC CGT TGG AGA TGG G-3′) were used to perform the 5′RACE. Primers L1 (5′-TCG CGG TGC TCG CCC ACG G-3′) and L2 (5′-CAC GGC TCA GGT TCC CTG GTC-3′) were used to perform the 3′RACE.

### Southern blotting

Fourteen micrograms of liver genomic DNA digested with *Eco*R I, *Eco*R V, *Bam*H I, *Xba* I, *Pst* I, *Hind* III, *Dra* I and *Pvu* II were fractionated in 0.9% agarose and transferred to Hybond N^+^ nylon membranes. Cμ-, Cα-, and Cυ-specific full-length as well as single-exon probes were labeled using a PCR digoxigenin probe synthesis kit (Roche, Germany). The primers used to amplify the full-length Cμ and Cμ4 exon probes were Cμs (5′- CGT CAA CGA CAG CAT CTC CA-3′), Cμas (5′-CAT TGA CCG AGG TGG GTT TA-3′), Cμ4s (5′-GCC AGA GCC CCG ACC ATC TAC-3′), and Cμ4as (5′- GAG GAC TTG TCC ACC GAC TTC-3′). The primers used to amplify the full-length Cα and Cα3 exon probes were Cαs (5′- CTG CGA CGA GGG AAA CGT CAC-3′), Cαas (5′-GAC GCT CAG CTT GCT GTA GAC-3′), Cα3s (5′-CTG CCC GTG GTC TCC ATC CTC-3′), and Cα3as (5′-AGT TCC TTC TGT GCA GAT TTG-3′). The primers used to amplify the full-length Cυ and Cυ4 exon probes were Cυs (5′- CTG CCT CAT CTC CCA CTT CTA C-3′), Cυas (5′- TTT ACC GGG GCT CCT GCT GAT-3′), Cυ4s (5′- CTG GCG CCC AGC GTC TAC CT-3′), and Cυ4as (5′- TGC GTT GCA CGA ACT TCA TG-3′). The hybridization and detection were performed following the manufacturer's hybridization instructions.

### PCR and Northern blotting detection of ostrich Ig gene expression in different tissues

The synthesized cDNA samples derived from RNA isolated from different organs (heart, liver, spleen, lung, kidney, large intestine, small intestine, and stomach) were used in RT-PCR to detect the expression of IgM, IgA, and IgY. The ostrich EIF1A1 gene was used as an internal control. The PCR primers were IgM-detections (5′-GGC CCC GTT GAT GTG GTG CCC A-3′); IgM-detectionAs (5′-GCT CGA AGC CGC ACT CCA G-3′); IgA-detections (5′-AAG AAC ATC GGG GAC TTA TG-3′); IgA-detectionAs (5′-GAC GCT CAG CTT GCT GTA GAC-3′); IgY-detections (5′-CTG CCT CAT CTC CCA CTT CTA C-3′); and IgY-detectionAs (5′-GAT GGT GCG TTG CAC GAA CTT-3′). Total RNA was used (7 µg/lane) for Northern analysis (the same kit as for the Southern blots). The primers used for probe amplification were the following: IgMs (5′-CCT GGA CCA ACT ACG TCA AC-3′); IgMas (5′-TTG CTC GTG TTC CTC ATC TC-3′); IgA1s (5′-CTG CGA CGA GGG AAA CGT CAC-3′); IgA1as (5′-GGA CAC TCG GCA AGC GAA CTC-3′); IgA2s (5′-GGA CCT CTA CCT CAG CCA GAA-3′); IgA2as (5′-GTG GAC GAA GGT GAT GGG GAT-3′); IgYs (5′-GAT CCA CGT CTT CGC CTT GC-3′); IgYas (5′-GAT GGT GCG TTG CAC GAA CTT-3′); IgLs (5′-GTG CAC CTC TTC CCT CCA TC-3′); IgLas (5′-TAG GAG GAG CAC TCG GAT CT-3′); EIF1A1s (5′-AAG GAG AAG ACC CAC ATC AAC-3′); and EIF1A1as (5′-GAG GAT CAT TCT TGC TGT CAC-3′).

### Western blotting

Membrane and cytosol proteins derived from different ostrich tissues were prepared using extraction kits (Beyotime, Beijing). Large-intestine secretions were diluted with 3% PBS. The oligopeptide that encodes ostrich IgA C_H_1–C_H_2 exons was synthesized, modified and coupled with KLH, and then intravenously injected into New Zealand rabbits. A polyclonal rabbit antiserum against the ostrich IgA C_H_1–C_H_2 domains was obtained and purified (Cwbiotech, Beijing). Samples were thermally denatured, separated by 12% SDS-PAGE and transferred to nitrocellulose membranes (Millipore, USA). The blot was blocked in Tris-buffered saline (TBS) containing 5% skim milk (w/v) for 1 h. Rabbit pAb and HRP-conjugated goat anti-rabbit IgG secondary antibodies (Cwbiotech, Beijing) were diluted using TBS+5% milk to 1∶600 and 1∶5000, respectively. The membranes were washed six times in TBS+0.05% Tween20 (TBST) between each step, and all incubations were performed at room temperature for 1 h. The bands were detected by incubation with Pierce ECL plus western blotting substrate (Thermo Fisher Scientific, USA) following the manufacturer's instructions.

### Sequence alignment and phylogenetic analysis

DNA and protein sequence editing, alignments, and comparisons were performed with the MegAlign software (DNASTAR). Phylogenetic trees were generated using MrBayes3.1.2 [50] and viewed in TreeView [51]. Multiple protein sequence alignments for the tree construction were performed using *ClustalW*. The accession numbers for the sequences (http://www.ncbi.nlm.nih.gov/sites/entrez) used for phylogenetic analysis are as follows: α or χ gene: chicken, S40610; cow, AF109167; duck, AJ314754; human, J00220; mouse, J00475; platypus, AY055778; *X. laevis*, BC072981; axolotl, AM774592; gecko, DQ523197; *X. tropicalis*, BC157650. δ gene: catfish, U67437; cow, AF411245; human, BC021276; mouse, J00449; *X. tropicalis,* DQ350886; lizard, EF690359. γ gene: human, J00228; mouse, J00453; platypus, AY055781; horse, AJ302055. ε gene: cow, AY221098; human, J00222; mouse, X01857; platypus, AY055780. μ gene: nurse shark, M92851; skate, M29679; catfish, X52617; lungfish, AF437724; zebrafish, AF281480; *X. laevis*, BC084123; axolotl, AM419754; lizard,EF690357; gecko, EU287911; chicken, X01613; duck, AJ314754; human, X14940; mouse, V00818; platypus, AY168639. υ gene: chicken, X07175; duck, X78273; *X. laevis*, X15114; lizard, EF690360; gecko, EU827594; *X. tropicalis*, BC089679; axolotl, X69492; Chinese soft-shell turtle, FJ605148. The accession numbers of sequences used for IgL constant regions are as follows; λ genes: chicken, X04768; duck, X82069; platypus, AF525122; human, J00252; mouse, AC14021; *X. laevis* type III, BC082898; *X. tropicalis* type III, BC121563; zebra finch, ACH44209; lizard IGIC1, IGIC2 (Ref.25); skate type II, L25566; sandbar shark type II, M81314; horn shark type III, L25561. κ genes: mouse, EF392842; human, AC210709; cow, BC122795; lizard (Ref.25); *X. laevis* BC068859; zebrafish IGIC1, AF246185; zebrafish IGIC3, AF246193; nurse shark NS4, L16765; carp IGIC1, AB015902; carp IGIC3, AB035730.σ genes: *X. laevis*, NM_001094414; *X. tropicalis*, AAH87749; zebrafish IGIC2, AF246162; nurse shark, EF114766; horn shark, EF114760; carp IGIC2 (AB091120). σ-cart genes: nurse shark NS5, AY720857; skate type, L25568; horn shark type I, X15316.

## Supporting Information

Figure S1 **Sequence alignment of the ostrich IgM C_H_ region with that of other species.** Dots are used to denote identical amino acids, and dashes are used to adjust the sequence alignment. Canonical cysteines are shaded and conserved N-linked glycosylation sites across species are in red. The alignment was performed using *ClustalW* with some manual adjustments.
(TIF)

Figure S2 **RT-PCR detection of the ostrich IgH gene expression in different tissues.**
(TIF)

Figure S3 **Sequence alignment of the ostrich IgA C_H_ region compared with that of other species.** The alignment was performed using the *ClustalW* method in MegAlign. Canonical cysteines are shaded, and conserved N-linked glycosylation sites across species are in red.
(TIF)

Figure S4 **Sequence of the short IgA membrane-bound form (VDJ-Cα1-Cα2-TM).**
(TIF)

Figure S5 **Sequence alignment of the ostrich IgY C_H_ region compared with that of other species.** The alignment was performed by using the *ClustalW* method in MegAlign. Canonical cysteines are shaded and conserved N-linked glycosylation sites across species are in red.
(TIF)

Figure S6 **Sequence alignment of the 54 CDR3.**
(TIF)

Figure S7 **Sequence alignment of the ostrich J_H_ gene segments.**
(TIF)

Figure S8 **Sequence alignment of the ostrich IgL constant region compared with that of other species.** The alignment was performed using the *ClustalW* method in MegAlign. Canonical cysteines are shaded.
(TIF)
