# Supplementary figures and images for: Analysis of Immunoglobulin Transcripts in the Ostrich Struthio camelus, a Primitive Avian Species
Source: PLoS One. 2012 Mar 29;7(3):e34346. doi: 10.1371/journal.pone.0034346 (PMC3315531; doi:10.1371/journal.pone.0034346)

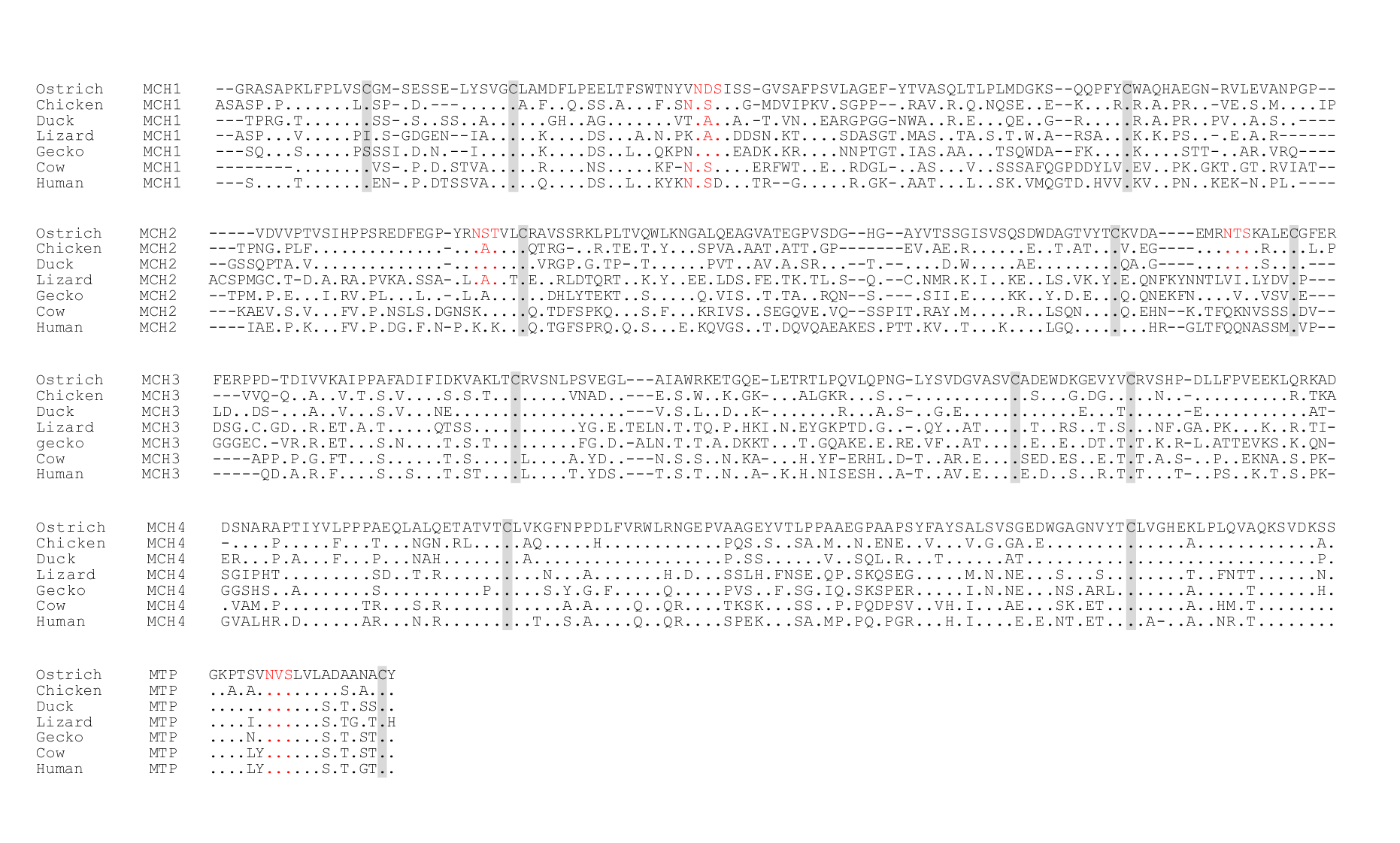

Supplement: Figure S1 — Sequence alignment of the ostrich IgM CH region with that of other species. Dots are used to denote identical amino acids, and dashes are used to adjust the sequence alignment. Canonical cysteines are shaded and conserved N-linked glycosylation sites across species are in red. The alignment was performed using ClustalW with some manual adjustments. (TIF) [file pone.0034346.s001.tif]

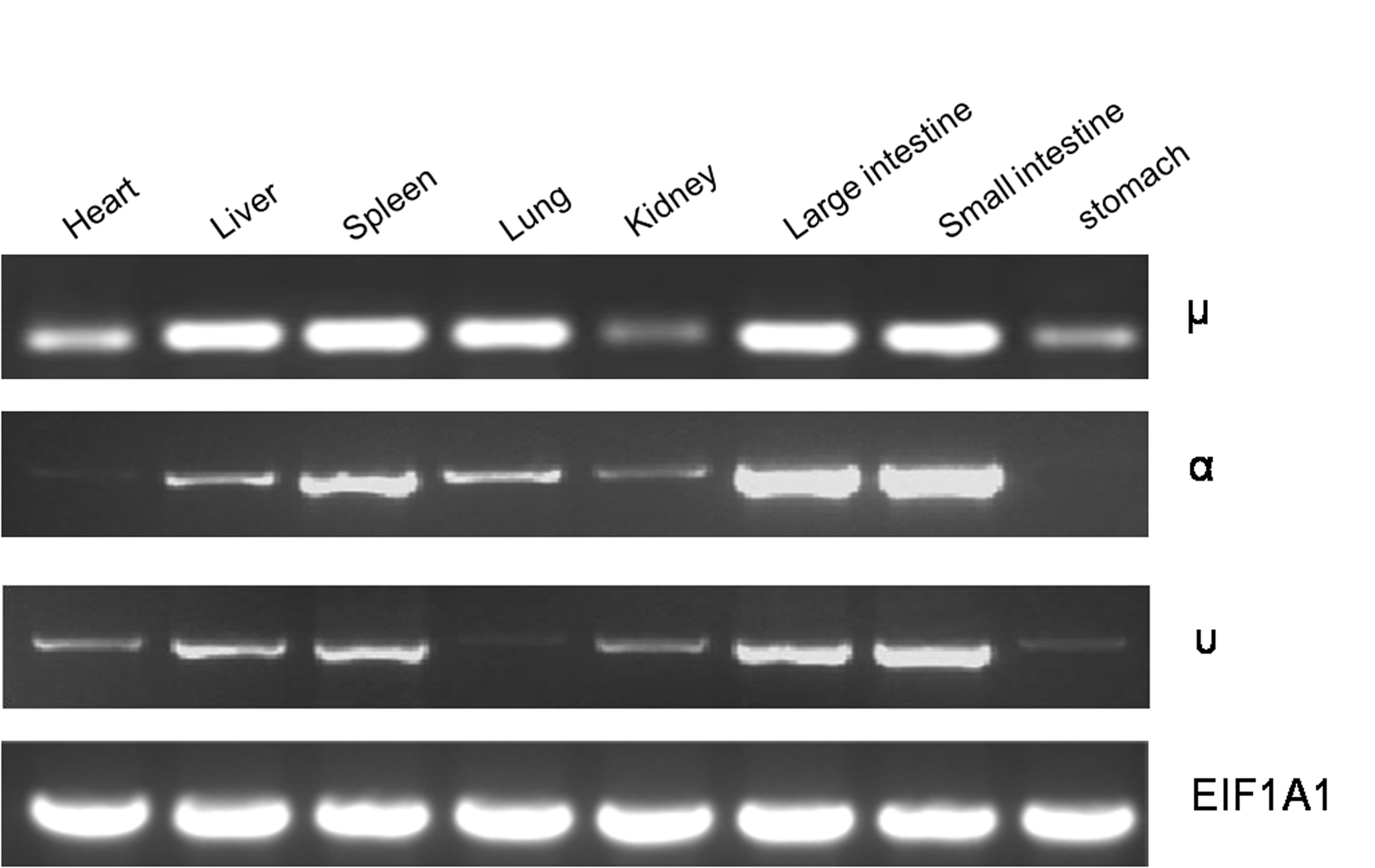

Supplement: Figure S2 — RT-PCR detection of the ostrich IgH gene expression in different tissues. (TIF) [file pone.0034346.s002.tif]

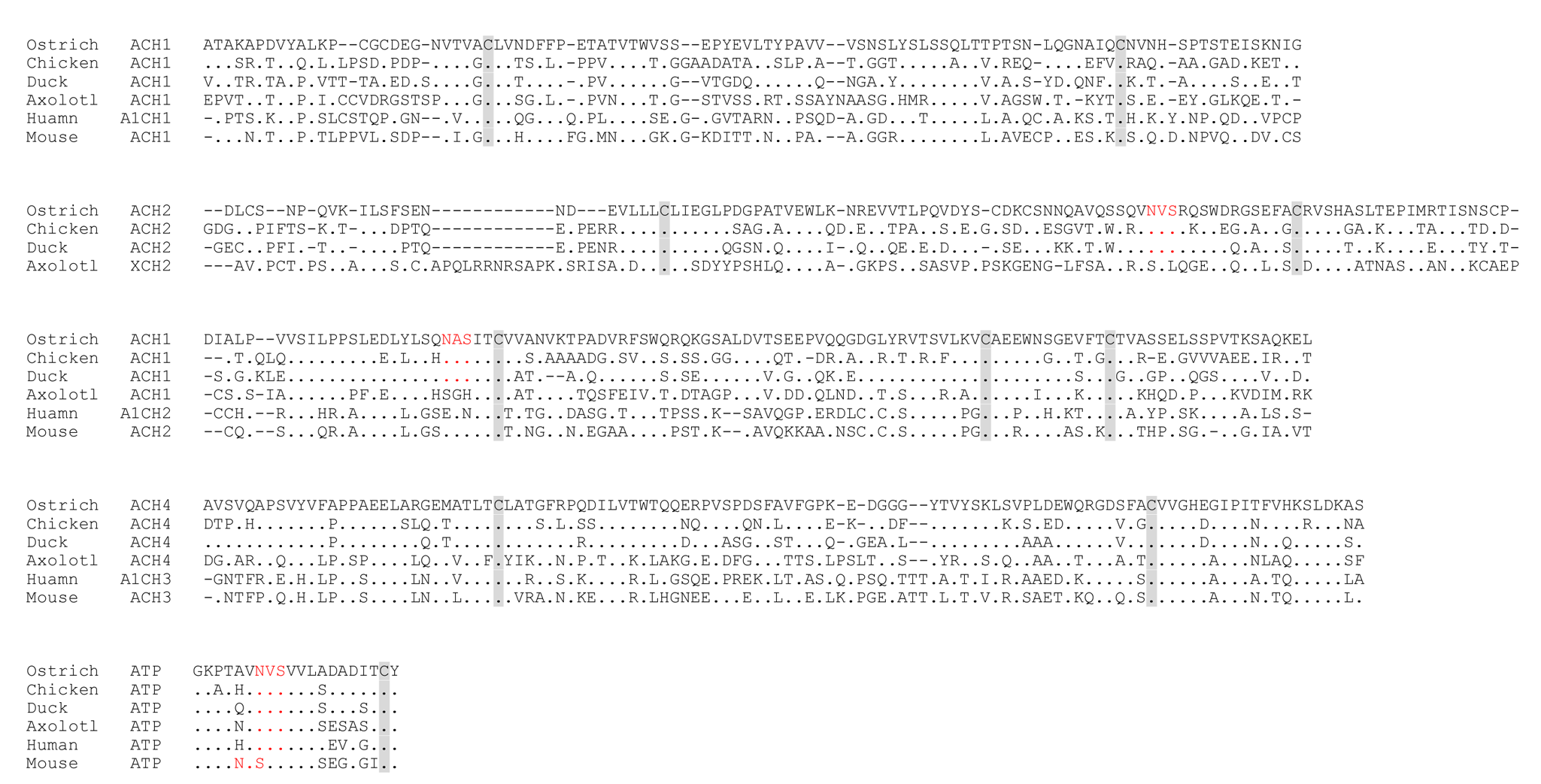

Supplement: Figure S3 — Sequence alignment of the ostrich IgA CH region compared with that of other species. The alignment was performed using the ClustalW method in MegAlign. Canonical cysteines are shaded, and conserved N-linked glycosylation sites across species are in red. (TIF) [file pone.0034346.s003.tif]

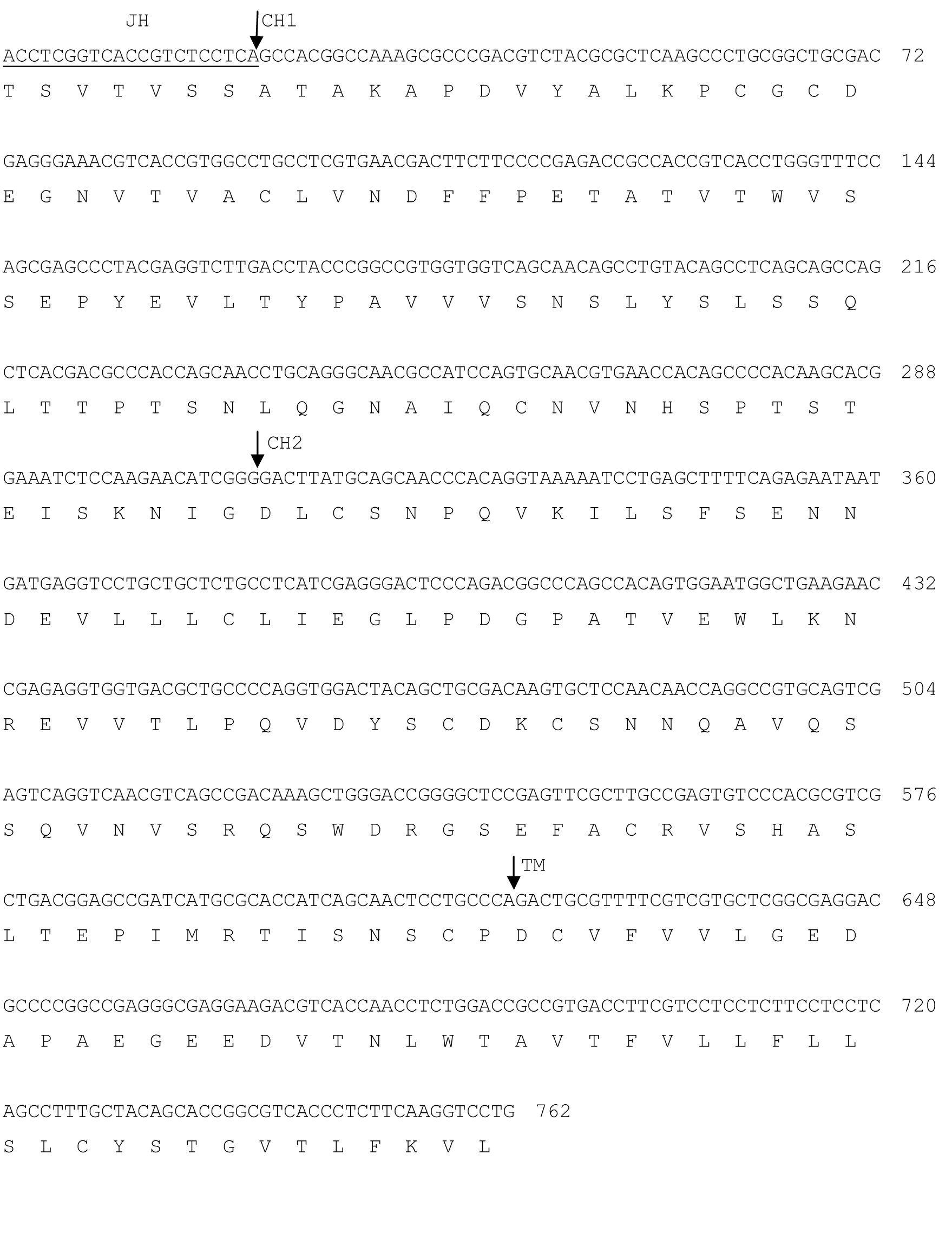

Supplement: Figure S4 — Sequence of the short IgA membrane-bound form (VDJ-Cα1-Cα2-TM). (TIF) [file pone.0034346.s004.tif]

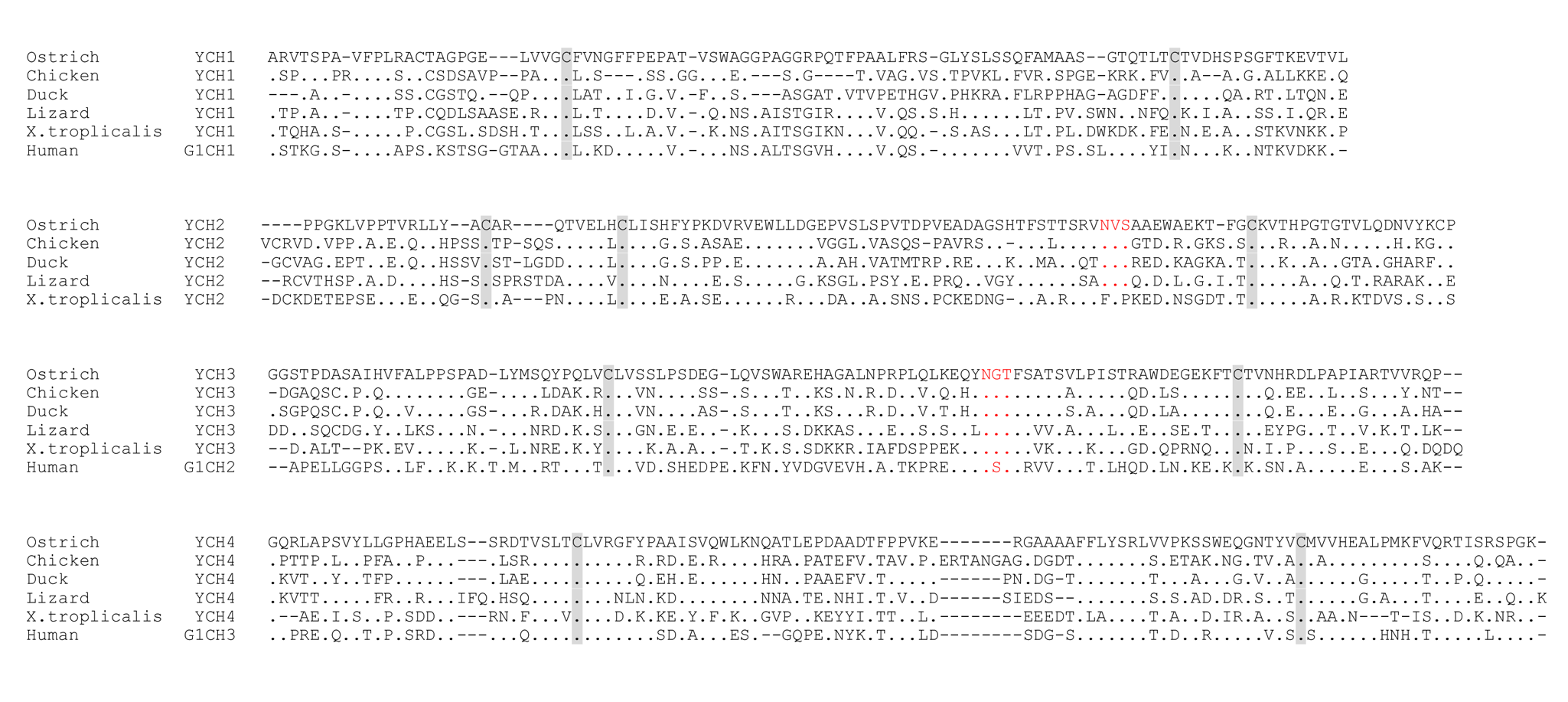

Supplement: Figure S5 — Sequence alignment of the ostrich IgY CH region compared with that of other species. The alignment was performed by using the ClustalW method in MegAlign. Canonical cysteines are shaded and conserved N-linked glycosylation sites across species are in red. (TIF) [file pone.0034346.s005.tif]

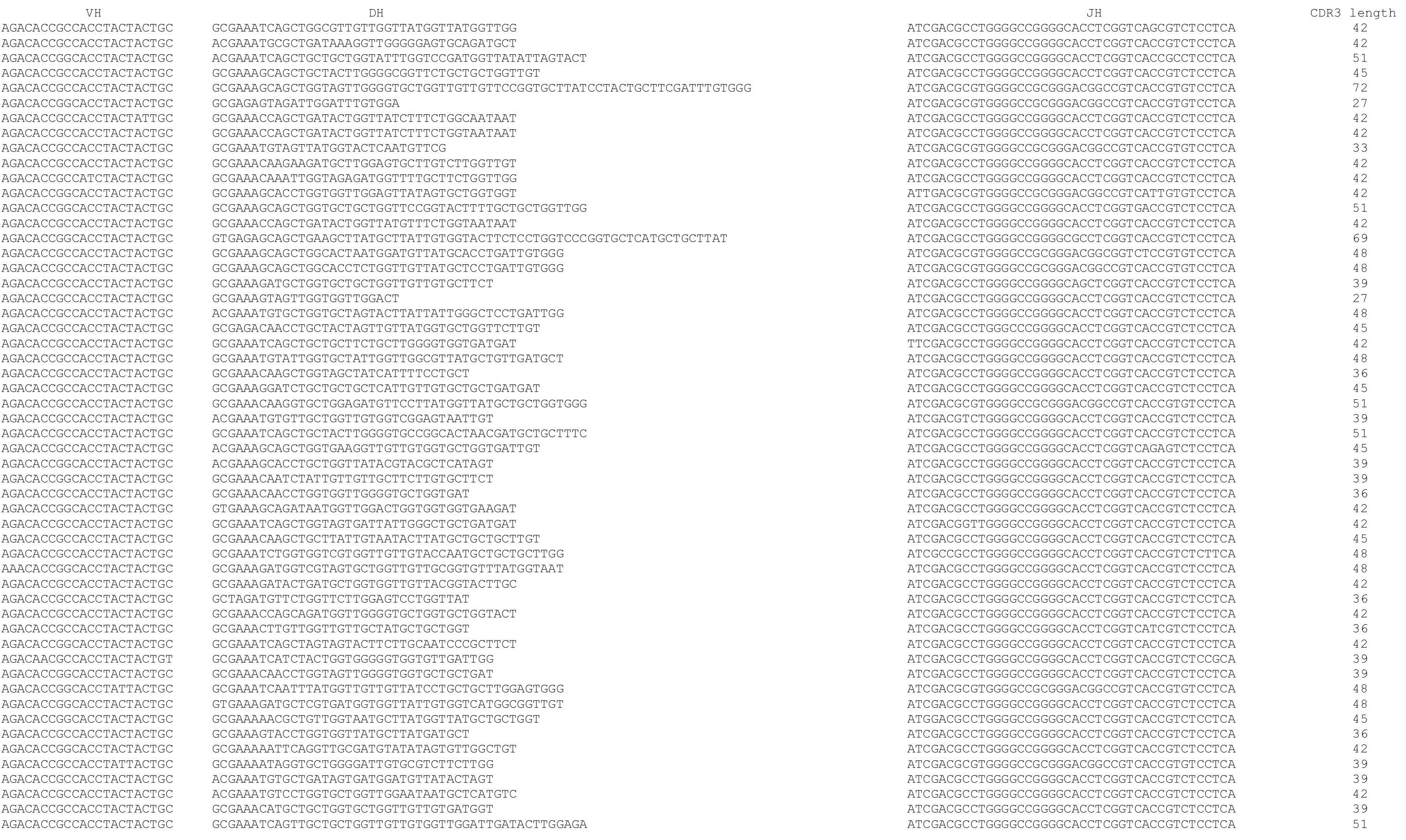

Supplement: Figure S6 — Sequence alignment of the 54 CDR3. (TIF) [file pone.0034346.s006.tif]

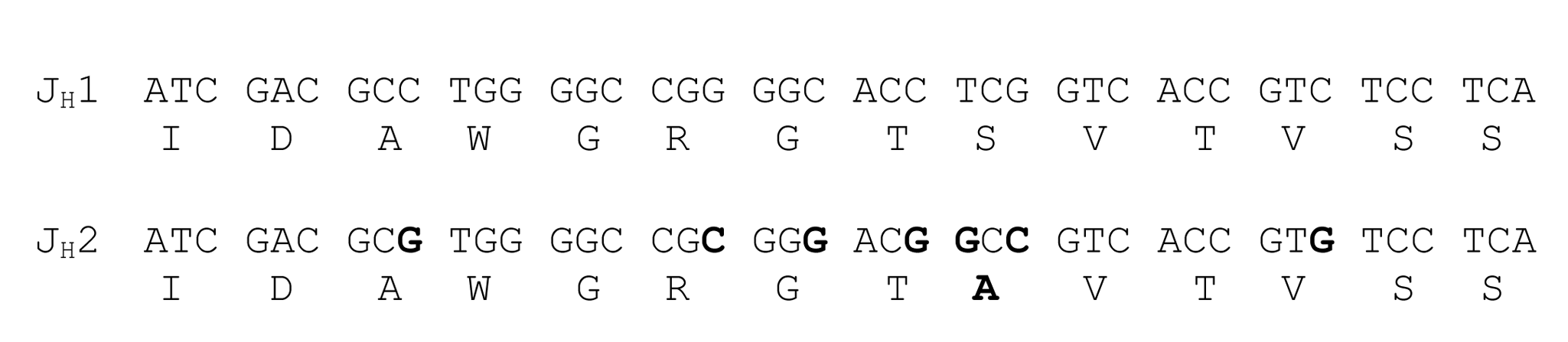

Supplement: Figure S7 — Sequence alignment of the ostrich JH gene segments. (TIF) [file pone.0034346.s007.tif]

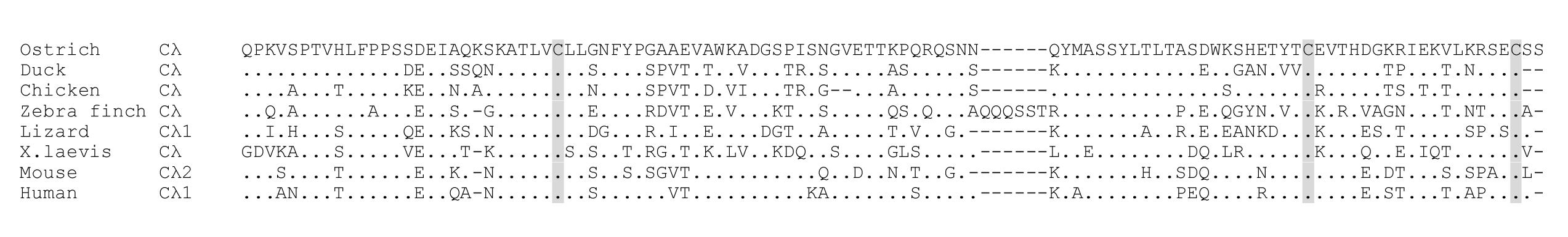

Supplement: Figure S8 — Sequence alignment of the ostrich IgL constant region compared with that of other species. The alignment was performed using the ClustalW method in MegAlign. Canonical cysteines are shaded. (TIF) [file pone.0034346.s008.tif]
